# Association of thyroid function, within the euthyroid range, with cardiovascular risk: The EPIPorto study

**DOI:** 10.3389/fendo.2022.1067801

**Published:** 2022-11-28

**Authors:** João Sérgio Neves, Ricardo Fontes-Carvalho, Marta Borges-Canha, Ana Rita Leite, Madalena von Hafe, Catarina Vale, Sandra Martins, João Tiago Guimarães, Davide Carvalho, Adelino Leite-Moreira, Ana Azevedo

**Affiliations:** ^1^ Departamento de Cirurgia e Fisiologia, Unidade de Investigação Cardiovascular, Faculdade de Medicina da Universidade do Porto, Porto, Portugal; ^2^ Department of Endocrinology, Diabetes and Metabolism, Centro Hospitalar Universitário de São João; Faculdade de Medicina da Universidade do Porto, Porto, Portugal; ^3^ Department of Cardiology, Centro Hospitalar Gaia/Espinho, Vila Nova de Gaia, Portugal; ^4^ Department of Clinical Pathology, Centro Hospitalar Universitário de São João, Porto, Portugal; ^5^ EPIUnit – Instituto de Saúde Pública, Universidade do Porto, Porto, Portugal; ^6^ Department of Biomedicine, Faculdade de Medicina da Universidade do Porto, Porto, Portugal; ^7^ Laboratório para a Investigação Integrativa e Translacional em Saúde Populacional (ITR), Porto, Portugal; ^8^ Instituto de Investigação e Inovação em Saúde (i3S), Universidade do Porto, Porto, Portugal; ^9^ Departamento de Ciências da Saúde Pública e Forenses e Educação Médica, Faculdade de Medicina da Universidade do Porto, Porto, Portugal

**Keywords:** thyroid, hypothyroidism, hyperthyroidism, cardiovascular risk, obesity, diabetes, dyslipidemia

## Abstract

**Background:**

Thyroid hormones are important modulators of cardiovascular function. Both hypothyroidism and hyperthyroidism are known to contribute to an increased cardiovascular risk. It remains uncertain whether thyroid hormones level within the euthyroid range are associated with cardiometabolic risk. We aimed to evaluate the association between thyroid function levels within the euthyroid range and cardiovascular risk in a population-based cohort.

**Methods:**

Eight hundred thirty-five subjects aged ≥45 years from the EPIPorto population-based cohort were included. We excluded participants with TSH, free T4 (FT4), or free T3 (FT3) outside of the reference range, or with previous cardiovascular or thyroid disease. The associations between thyroid function, cardiovascular risk factors and the 10-year estimated risk of cardiovascular events (using SCORE2 and SCORE2-OP) were evaluated in linear and logistic regression models, crudely and adjusting for age, sex, BMI, diabetes, and smoking.

**Results:**

The mean age of the participants was 61.5 (SD 10.5) years and 38.9% were men. Eleven percent of the participants had diabetes, 47.8% had dyslipidemia, and 54.8% had hypertension. The mean body mass index (BMI) was 27.4 (SD 4.6) kg/m^2^, and the median (percentile25-75) 10-year risk of cardiovascular events was 5.46% (2.92, 10.11). Participants with higher BMI, larger waist circumference and higher hs-CRP had higher levels of FT3 and FT3/FT4 ratio. Lower FT3/FT4 ratio and higher FT4 levels were associated with higher prevalence of diabetes and more adverse lipid profile. Higher TSH, lower FT3 and lower FT3/FT4 ratio were associated with lower eGFR. Lower FT3, lower FT3/FT4 ratio and higher FT4 were associated with an increased 10-year risk of cardiovascular events.

**Conclusions:**

In a population-based study, variations of thyroid function within the euthyroid range were associated with cardiovascular risk factors. On one hand, individuals with higher BMI, larger waist circumference and higher hs-CRP had higher levels of FT3 and FT3/FT4 ratio. On the other hand, a decreased conversion of T4 to T3 (lower FT3, lower FT3/FT4 ratio and/or higher FT4) was associated with a higher prevalence of diabetes, a more adverse lipid profile, a lower eGFR and an increased 10-year risk of cardiovascular events.

## Introduction

Thyroid hormones have a central role on cardiovascular homeostasis. Thyroid hormones modulate cardiac and vascular function, basal metabolic rate, glucose tolerance, serum lipids and kidney function (1–3). Both overt hypothyroidism and hyperthyroidism are known to contribute to a higher risk of ischemic heart disease, stroke and heart failure (4–6). Patients with hypothyroidism also have an increased prevalence of several cardiovascular risk factors, such as dyslipidemia, hypertension, obesity and diabetes (5, 7, 8). Patients with hyperthyroidism have an increased prevalence of hypertension and may have a hypercoagulable state (1, 9). A large body of evidence suggests that also subclinical hypo- and hyperthyroidism contribute to increased cardiovascular risk (10, 11). From a physiological perspective, it is likely that there is a continuum of effects across the spectrum of thyroid function, including the euthyroid range. However, while the effects of overt and subclinical thyroid dysfunction on cardiovascular risk factors have been well characterized (1, 12), fewer studies addressed the association of thyroid hormones within the euthyroid range with cardiometabolic risk. In a study of US community-dwelling older adults, lower TSH and higher free thyroxine (FT4) within the euthyroid range were associated with an increased risk of adverse events, including mortality (13). In the Rotterdam study, in elderly subjects, high-normal FT4 and low-normal TSH were associated with an increased risk of atrial fibrillation (14). On the other hand, in a study from Groningen (Netherlands), in people aged 28–75 years, low normal FT4 levels were significantly associated with adverse lipid profile and increased insulin resistance (15). Higher TSH levels and lower FT4 levels were also associated with increased risk of incident diabetes, especially in individuals with prediabetes (16). Lower free triiodothyronine (FT3) levels have been associated with increased risk of cardiovascular mortality in the general population (17) and in patients with established cardiovascular disease (18–20). In heart failure, low normal FT3 have been associated with higher BNP levels and with increased risk of adverse events (21, 22).

The EPIPorto study is a population-based study (23) that includes detailed data on cardiovascular risk factors. In the second evaluation of the cohort, participants aged 45 years or older were included, an age group that is particularly relevant as it represents the population usually targeted by cardiovascular preventive interventions. Therefore, in this analysis of the EPIPorto study, we aimed to evaluate the association of thyroid function levels within the euthyroid range with cardiovascular risk in middle-aged and elderly individuals.

## Methods

### Study design and participants

The EPIPorto study is a population-based cohort (23), ongoing for more than 20 years, with the main objective of assessing the determinants of health in the adult population of Porto, Portugal. In the baseline evaluation (1999-2003), 2485 persons were selected at random and have been evaluated over time. Between October 2006 and July 2008, participants with 45 years of age or older over were eligible to an evaluation that included cardiovascular history, physical examination, anthropometric evaluation, collection of fasting blood sample and a transthoracic echocardiogram. Among 2048 cohort members in the eligible age range at this time, 134 (6.5%) had died, 198 (9.7%) refused re-evaluation and 580 (28.3%) were lost to follow-up. We further excluded 301 patients with history of heart disease (significant valvular heart disease, prior cardiac surgery, previous myocardial infarction, percutaneous or surgical revascularization; n=136), history of thyroid disease (n=50), treatment with drugs that interfere with thyroid function (n=17); without serum available for thyroid function determination (n=40); or with TSH, free thyroxine (FT4) or free triiodothyronine (FT3) outside of the reference range (0.35-4.94 μIU/mL for TSH, 0.70-1.48 ng/dL for FT4 and 1.71-3.71 pg/mL for FT3) (n=58). Eight hundred and thirty-five participants were included in our analysis. This study was approved by the ethics committee of Centro Hospitalar Universitário São João/Faculdade de Medicina da Universidade do Porto.

### Thyroid function measurements

TSH, FT3, and FT4 were measured in serum samples stored at -80°C by an electrochemiluminescence immunoassay using an Abbott Architect i2000 analyzer (Abbott Diagnostics). Previous studies have shown that TSH and thyroid hormones can be analyzed reliably in frozen stored samples (24). The FT3/FT4 ratio, an indicator of peripheral deiodinase activity, was calculated dividing plasma concentrations of FT3 by FT4 levels (25).

### Anthropometric and analytical measurements

Anthropometric measures (body mass index, waist circumference and waist-to-hip circumference ratio) were performed after an overnight fast, with the participant wearing light clothing and no footwear. Body weight was measured to the nearest 0.1 kg using a digital scale, and height was measured to the nearest centimeter in the standing position using a wall stadiometer. Body mass index (BMI) was calculated as weight in kilograms divided by squared height in meters. Body composition was assessed by bioelectrical impedance analysis (Tanita Corp, Arlington Heights, IL) to determine fat mass. Body fat percentage was calculated as (fat mass/body weight) x 100. Fat mass index was calculated as fat mass in kilograms divided by squared height in meters.

Blood pressure was determined by two measurements separated by at least 5 minutes after a 10-minute rest. When the difference between measurements was larger than 5 mmHg for systolic or diastolic blood pressure a third measurement was taken, and the mean of the 2 closest values was used.

Lipid profile (total cholesterol, LDL cholesterol, HDL cholesterol and triglycerides), glucose profile (fasting glucose and homeostatic model assessment for insulin resistance [HOMA-IR]), adipokines (leptin and adiponectin), creatinine and high-sensitivity C-reactive protein (hs-CRP) were obtained from a fasting venous blood sample in the morning. Adipokines were only evaluated in a subsample of participants (leptin in 445 participants and adiponectin in 441 participants). HOMA-IR was calculated according to the formula: fasting insulin (µU/L) x fasting glucose (mg/dL)/405 (26). Estimated glomerular filtration rate (eGFR) was estimated using Chronic Kidney Disease Epidemiology Collaboration (CKD-EPI) formula (27).

### Comorbidities and clinical definitions

Obesity was defined as BMI ≥30 kg/m^2^. Hypertension was defined as systolic blood pressure ≥140 mmHg or diastolic blood pressure ≥90 mmHg at the time of the visit or use of antihypertensive drugs (28). Diabetes was defined as fasting blood glucose ≥126 mg/dL, self-reported history of diabetes, or use of diabetes medications (29). Dyslipidemia was defined as low-density lipoprotein cholesterol ≥160 mg/dL, triglycerides ≥200 mg/dL, high-density lipoprotein cholesterol <40 mg/dL, or use of lipid-lowering drugs (30). Chronic kidney disease (CKD) was defined as eGFR <60 mL/min/1.73 m^2^ (31). Risk of cardiovascular events (10 year risk of fatal and non-fatal events) was calculated using SCORE2 (Systematic Coronary Risk Estimation 2) (32), for participants aged 40-69 years, and SCORE2-OP (Systematic Coronary Risk Estimation 2-Older Persons) (33), for participants aged over 70 years or older. Cardiovascular disease risk categories based on SCORE2 and SCORE2-OP were used according to the European Society of Cardiology (ESC) classification: low-moderate risk (<50 years: risk <2.5%; 50–69 years: <5%; ≥70 years: <7.5%), high risk (<50 years: 2.5-7.5%; 50–69 years: 5-10%; ≥70 years: 7.5-15%) or very high risk (<50 years: ≥7.5%; 50–69 years: ≥10%; ≥70 years: ≥15%) (34).

### Statistical analysis

Linear regression models were used to evaluate the associations of TSH, FT3, FT4 and FT3/FT4 ratio (independent variables) and anthropometric parameters, glucose profile, lipid profile, adipokines, hs-CRP and kidney function (dependent variables). The assumptions of normality, homoscedasticity, and linearity were assessed using the Q-Q plot of residuals, plot of residuals against predicted values and plots of residuals against each variable in the regression models. Logistic regression models were used to evaluate the associations of TSH, FT3, FT4 and FT3/FT4 ratio (independent variables) and diabetes, obesity, dyslipidemia, hypertension and CKD (dependent variables). These associations were evaluated crudely and in models adjusted for sex, age and current smoking (model 1). Sex and age were included in the adjustment model due to their known association with thyroid function and their association with cardiovascular risk factors. Smoking is also strongly associated with several cardiovascular risk factors and influences thyroid hormone levels. Associations were also evaluated in a model adjusted for sex, age, current smoking, BMI and diabetes (model 2). This model was used to evaluate if the association of thyroid function with cardiovascular risk factors was independent of BMI and diabetes. Although thyroid hormones may influence BMI and the risk of diabetes, BMI and diabetes also significantly affect thyroid hormone levels and are potential confounders of the association of thyroid hormones with the cardiovascular risk (35). Models evaluating the association of thyroid function with anthropometric parameters did not include BMI; models evaluating the association of thyroid function with lipid profile and dyslipidemia were restricted to participants not treated with lipid-lowering drugs; and models evaluating the association with glucose profile were restricted to participants not treated with antidiabetic drugs. In models evaluating the association of thyroid function with 10-year risk of cardiovascular events, participants with diabetes were excluded as SCORE2 and SCORE2-OP do not apply to this group of patients. These models were adjusted only for BMI, because sex, age and smoking are part of the model used to calculated SCORE2 and SCORE2-OP. We also evaluated the distribution of cardiovascular disease risk categories according to terciles of TSH, FT4, FT3 and FT3/FT4 ratio. The association of thyroid function parameters (independent variable) with cardiovascular disease risk categories (dependent variable) was evaluated using ordered logistic regression.

Continuous variables are presented as mean (standard deviation) for continuous normally distributed variables, and as median (percentile 25 – percentile 75) for non-normally distributed continuous variables. TSH, triglycerides, HOMA-IR, hs-CRP, adiponectin, leptin and the 10-year risk of cardiovascular events were log-transformed for inclusion in regression models due to skewness. Statistical analyses were performed with Stata software, version 17.0 (StataCorp).

## Results

A total of 835 participants were included in this analysis. The mean age of participants was 61.5 (SD 10.5) years, 38.9% were male, the mean BMI was 27.4 (SD 4.6) kg/m^2^, 11.4% had diabetes, 47.8% had dyslipidemia, 54.8% had hypertension, 10.6% had CKD, and 14.7% were current smokers (**Table 1**). The median 10-year risk of fatal and non-fatal cardiovascular events was 5.5% (percentile 25-75, 2.9-10.1).

**Table 1:** Characteristics of the study sample (n=835).

Male sex, %	325 (38.9%)
Age, years	61.5 ± 10.5
Hypertension, %	455 (54.8%)
Dyslipidemia, %	396 (47.8%)
Obesity, %	210 (25.2%)
Diabetes, %	95 (11.4%)
CKD, %	88 (10.6%)
Current smoking, %	123 (14.7%)
Body mass index, kg/m^2^	27.4 ± 4.6
Waist circumference, cm	92.8 ± 11.4
Waist-to-hip ratio	0.9 ± 0.1
Body fat percentage, %	31.0 ± 8.5
Systolic pressure, mmHg	132.9 ± 19.6
Diastolic pressure, mmHg	78.3 ± 11.3
TSH, μIU/mL	1.2 (0.9, 1,7)
FT4, ng/dL	1.1 ± 0.1
FT3, pg/mL	2.5 ± 0.4
FT3/FT4 ratio	2.4 ± 0.5
Total cholesterol, mg/dL	219.3 ± 39.0
LDL cholesterol, mg/dL	133.2 ± 34.5
HDL cholesterol, mg/dL	59.7 ± 13.1
Triglycerides, mg/dL	113.0 (84.0, 157.0)
eGFR, mL/min/1.73 m^2^	77.8 ± 14.5
Fasting plasma glucose, mg/dL	101.9 ± 23.9
HOMA-IR	1.1 (0.6, 1.8)
Hs-CRP, mg/dL	0.2 (0.1, 0.4)
Adiponectin, ng/ml	6249.5 (3293.1, 10249.5)
Leptin, ng/ml	14.0 (8.0, 22.8)
10-year risk of cardiovascular events, %	5.46 (2.92, 10.11)

Categorical variables are presented as counts (percentages). Continuous variables are presented as mean ± standard deviation or median (25^th^, 75^th^ percentile). 10-year risk of fatal and non-fatal cardiovascular events was calculated using SCORE2 (Systematic Coronary Risk Estimation 2) and SCORE2-OP (Systematic Coronary Risk Estimation 2-Older Persons). CKD, chronic kidney disease; eGFR, Estimated glomerular filtration rate; Hs-CRP, high sensitivity C-reactive protein; HOMA-IR, Homeostatic Model Assessment for Insulin Resistance.

Thyroid hormone levels were not significantly associated with the diagnosis of obesity (**Table 2**). On the other hand, FT3 was positively associated with BMI and fat mass index, and both FT3 and FT3/FT4 ratio were positively associated with waist circumference in adjusted models. Additionally, TSH was positively associated with leptin in unadjusted and adjusted models, and the FT3/FT4 ratio was positively associated with leptin in unadjusted model and in model 1 (adjusted for sex, age and current smoking), but not in model 2 (further adjusted for BMI and diabetes). There were no associations of thyroid function with adiponectin levels (**Table 2**). FT3 and FT3/FT4 ratio were positively associated with hs-CRP in unadjusted and adjusted models (**Table 2**).

**Table 2:** Association of thyroid function with obesity, anthropometric parameters, plasma adipokines and high-sensitivity C-reactive protein.

	TSH (per μIU/mL)		FT4 (per ng/dL)		FT3 (per pg/mL)		FT3/FT4 ratio (per unit)		
	Odds ratio(95% CI)	P Value	Odds ratio(95% CI)	P Value	Odds ratio(95% CI)	P Value	Odds ratio(95% CI)		P Value
**Outcomes**									
Obesity									
Unadjusted	1.38 (0.99-1.91)	0.057	0.77 (0.23-2.60)	0.671	1.36 (0.90-2.05)	0.140		1.32 (0.93-1.85)	0.116
Model 1	1.16 (0.83-1.63)	0.388	0.81 (0.24-2.81)	0.744	1.38 (0.91-2.11)	0.133		1.31 (0.92-1.87)	0.137
Model 2	1.16 (0.83-1.63)	0.389	0.69 (0.20-2.42)	0.565	1.43 (0.93-2.19)	0.103		1.37 (0.96-1.95)	0.087
	**TSH (per μIU/mL)**		**FT4 (per ng/dL)**		**FT3 (per pg/mL)**			**FT3/FT4 ratio (per unit)**	
	**β (95% CI)**	**P Value**	**β (95% CI)**	**P value**	**β (95% CI)**	**P Value**		**β (95% CI)**	**P Value**
**Outcomes**									
BMI, kg/m^2^									
Unadjusted	0.36 (-0.30 to 1.02)	0.289	-0.21 (-2.89 to 2.47)	0.876	**0.85 (0.04 to 1.67)**	**0.041**		0.67 (-0.14 to 1.49)	0.104
Model 1	-0.01 (-0.65 to 0.63)	0.985	-0.13 (-2.72 to 2.45)	0.921	**0.90 (0.12 to 1.68)**	**0.024**		0.68 (-0.11 to 1.47)	0.092
Model 2	-0.01 (-0.65 to 0.63)	0.978	-0.52 (-3.08 to 2.05)	0.692	**0.96 (0.18 to 1.74)**	**0.016**		0.78 (-0.00 to 1.56)	0.051
Waist circumference, cm									
Unadjusted	-0.22 (-1.90 to 1.46)	0.796	0.23 (-6.02 to 6.48)	0.942	1.13 (-0.87 to 3.14)	0.268		0.81 (-0.98 to 2.61)	0.374
Model 1	-0.10 (-1.68 to 1.47)	0.897	-0.64 (-6.83 to 5.56)	0.840	**1.89 (0.00 to 3.78)**	**0.050**		1.48 (-0.28 to 3.25)	0.100
Model 2	-0.13 (-1.69 to 1.44)	0.873	-1.83 (-7.89 to 4.23)	0.554	**2.07 (0.18 to 3.96)**	**0.032**		**1.78 (0.05 to 3.51)**	**0.044**
Waist-to-hip ratio									
Unadjusted	**-0.02 (-0.03 to -0.01)**	**0.004**	0.03 (-0.01 to 0.07)	0.211	0.00 (-0.01 to 0.02)	0.760		-0.00 (-0.01 to 0.01)	0.557
Model 1	-0.01 (-0.02 to 0.00)	0.085	0.02 (-0.02 to 0.05)	0.417	0.01 (-0.00 to 0.02)	0.074		0.00 (-0.01 to 0.01)	0.376
Model 2	-0.01 (-0.02 to 0.00)	0.073	0.01 (-0.03 to 0.04)	0.662	**0.01 (0.00 to 0.02)**	**0.046**		0.01 (-0.00 to 0.02)	0.206
Fat percentage, %									
Unadjusted	**1.97 (0.78 to 3.16)**	**0.001**	0.89 (-3.80 to 5.58)	0.710	0.86 (-0.65 to 2.37)	0.262		0.46 (-0.87 to 1.79)	0.497
Model 1	0.35 (-0.60 to 1.29)	0.472	0.74 (-2.89 to 4.36)	0.690	1.07 (-0.10 to 2.23)	0.073		0.55 (-0.53 to 1.63)	0.316
Model 2	0.34 (-0.61 to 1.29)	0.482	0.38 (-3.24 to 4.00)	0.836	1.13 (-0.04 to 2.30)	0.058		0.64 (-0.43 to 1.72)	0.238
Fat mass index, kg/m^2^									
Unadjusted	**0.75 (0.24 to 1.27)**	**0.004**	-0.11 (-2.30 to 2.07)	0.918	0.53 (-0.11 to 1.18)	0.103		0.43 (-0.21 to 1.06)	0.191
Model 1	0.19 (-0.27 to 0.65)	0.413	-0.10 (-2.01 to 1.81)	0.920	**0.59 (0.04 to 1.14)**	**0.037**		0.44 (-0.13 to 1.01)	0.133
Model 2	0.19 (-0.27 to 0.64)	0.424	-0.35 (-2.26 to 1.57)	0.720	**0.63 (0.08 to 1.19)**	**0.026**		0.50 (-0.07 to 1.07)	0.084
Leptin, ng/ml									
Unadjusted	**0.39 (0.23 to 0.55)**	**<0.001**	-0.43 (-1.05 to 0.20)	0.180	**0.23 (0.02 to 0.44)**	**0.032**		**0.25 (0.07 to 0.42)**	**0.005**
Model 1	**0.30 (0.17 to 0.43)**	**<0.001**	-0.26 (-0.79 to 0.26)	0.321	0.19 (-0.00 to 0.38)	0.051		**0.17 (0.01 to 0.34)**	**0.039**
Model 2	**0.25 (0.14 to 0.35)**	**<0.001**	-0.18 (-0.64 to 0.28)	0.434	0.11 (-0.05 to 0.28)	0.181		0.10 (-0.05 to 0.24)	0.191
Adiponectin, ng/ml									
Unadjusted	0.06 (-0.11 to 0.22)	0.501	0.39 (-0.21 to 0.98)	0.201	-0.01 (-0.24 to 0.21)	0.901		-0.10 (-0.31 to 0.11)	0.369
Model 1	0.04 (-0.12 to 0.21)	0.609	0.44 (-0.15 to 1.04)	0.143	-0.03 (-0.26 to 0.20)	0.824		-0.12 (-0.33 to 0.09)	0.258
Model 2	0.06 (-0.10 to 0.23)	0.443	0.48 (-0.11 to 1.08)	0.113	-0.02 (-0.25 to 0.21)	0.869		-0.12 (-0.33 to 0.09)	0.262
Hs-CRP, mg/dL									
Unadjusted	0.08 (-0.07 to 0.24)	0.298	0.19 (-0.39 to 0.78)	0.511	**0.28 (0.08 to 0.47)**	**0.006**		**0.17 (0.00 to 0.33)**	**0.048**
Model 1	0.07 (-0.09 to 0.23)	0.387	0.09 (-0.49 to 0.67)	0.758	**0.34 (0.14 to 0.54)**	**0.001**		**0.23 (0.06 to 0.40)**	**0.007**
Model 2	0.07 (-0.08 to 0.22)	0.342	0.06 (-0.48 to 0.60)	0.822	**0.27 (0.09 to 0.46)**	**0.003**		**0.19 (0.03 to 0.34)**	**0.020**

Model 1: age, sex. Model 2: age, sex, BMI, diabetes, smoking (BMI not included in model for obesity). Linear regression models were used to evaluate the associations of TSH, FT3, FT4 and FT3/FT4 ratio (independent variables) and anthropometric parameters, adipokines, hs-CRP and (dependent variables). Logistic regression models were used to evaluate the associations of TSH, FT3, FT4 and FT3/FT4 ratio (independent variables) and obesity. TSH, hs-CRP, adiponectin, and leptin were log-transformed. BMI, body mass index; Hs-CRP, high sensitivity C-reactive protein; TSH, Thyroid-stimulating hormone; FT4, free thyroxine; FT3, free triiodothyronine. Significant associations are highlighted in bold.

Regarding the association of thyroid function with diabetes, higher levels of FT4 and lower ratio of FT3/FT4 were associated with a higher odds of diabetes (**Table 3**). Among participants not treated with antidiabetic drugs, FT3 was positively associated with HOMA-IR in model 1 but not after further adjustment in model 2 (**Table 3**).

**Table 3:** Association of thyroid function with diabetes and glucose profile.

	TSH (per μIU/mL)		FT4 (per ng/dL)		FT3 (per pg/mL)		FT3/FT4 ratio (per unit)	
	Odds ratio(95% CI)	P Value	Odds ratio(95% CI)	P Value	Odds ratio(95% CI)	P Value	Odds ratio(95% CI)	P Value
**Outcomes**								
Diabetes								
Unadjusted	1.04 (0.67-1.63)	0.857	**9.85 (1.91-50.85)**	**0.006**	0.59 (0.33-1.05)	0.071	**0.48 (0.29-0.80)**	**0.005**
Model 1	1.02 (0.64-1.62)	0.925	**7.05 (1.35-36.67)**	**0.020**	0.71 (0.40-1.28)	0.252	**0.58 (0.34-0.97)**	**0.038**
Model 2	1.01 (0.63-1.62)	0.956	**6.99 (1.30-37.40)**	**0.023**	0.64 (0.35-1.17)	0.144	**0.53 (0.31-0.91)**	**0.020**
	**TSH (per μIU/mL)**		**FT4 (per ng/dL)**		**FT3 (per pg/mL)**		**FT3/FT4 ratio (per unit)**	
	**β (95% CI)**	**P Value**	**β (95% CI)**	**P Value**	**β (95% CI)**	**P Value**	**β (95% CI)**	**P Value**
**Outcomes**								
Fasting plasma glucose, mg/dL								
Unadjusted model	-0.12 (-2.49 to 2.26)	0.922	4.63 (-4.38 to 13.64)	0.314	1.05 (-1.92 to 4.01)	0.489	-0.17 (-2.71 to 2.37)	0.895
Model 1	0.46 (-1.89 to 2.81)	0.702	2.52 (-6.33 to 11.36)	0.577	2.35 (-0.59 to 5.29)	0.117	1.12 (-1.42 to 3.65)	0.387
Model 2	0.71 (-1.48 to 2.90)	0.524	-0.11 (-8.38 to 8.16)	0.979	1.83 (-0.92 to 4.58)	0.192	1.18 (-1.19 to 3.55)	0.329
HOMA-IR								
Unadjusted model	-0.08 (-0.22 to 0.05)	0.223	0.07 (-0.44 to 0.58)	0.788	0.17 (-0.00 to 0.33)	0.053	0.10 (-0.04 to 0.25)	0.158
Model 1	-0.09 (-0.23 to 0.04)	0.174	0.08 (-0.43 to 0.59)	0.764	**0.19 (0.02 to 0.36)**	**0.027**	0.12 (-0.02 to 0.26)	0.105
Model 2	-0.07 (-0.20 to 0.05)	0.255	0.06 (-0.41 to 0.53)	0.803	0.11 (-0.04 to 0.27)	0.156	0.07 (-0.06 to 0.20)	0.300

Model 1: age, sex. Model 2: age, sex, BMI, diabetes, smoking (diabetes not included in model for diabetes). Analysis of fasting plasma glucose and HOMA-IR restricted to participants not treated with antidiabetic drugs. Linear regression models were used to evaluate the associations of TSH, FT3, FT4 and FT3/FT4 ratio (independent variables) and glucose profile (dependent variables). Logistic regression models were used to evaluate the associations of TSH, FT3, FT4 and FT3/FT4 ratio (independent variables) and diabetes (dependent variables). TSH and HOMA-IR were log-transformed. HOMA-IR, Homeostatic Model Assessment for Insulin Resistance; TSH, Thyroid-stimulating hormone; FT4, free thyroxine; FT3, free triiodothyronine. Significant associations are highlighted in bold.

In respect to the lipid profile, thyroid function was not associated with the odds of dyslipidemia (**Table 4**). Among participants not treated with lipid-lowering drugs, FT4 was positively associated with total cholesterol, LDL cholesterol and triglycerides, and FT3/FT4 ratio was negatively associated with total cholesterol and LDL cholesterol. No significant associations with HDL were observed (**Table 4**).

**Table 4:** Association of thyroid function with dyslipidemia and lipid profile.

	TSH (per μIU/mL)		FT4 (per ng/dL)		FT3 (per pg/mL)		FT3/FT4 ratio (per unit)	
	Odds ratio(95% CI)	P Value	Odds ratio(95% CI)	P Value	Odds ratio(95% CI)	P Value	Odds ratio(95% CI)	P Value
**Outcomes**								
Dyslipidemia								
Unadjusted	1.10 (0.82-1.46)	0.531	2.45 (0.85-7.09)	0.097	0.97 (0.68-1.39)	0.881	0.81 (0.60-1.09)	0.168
Model 1	1.15 (0.86-1.55)	0.345	1.95 (0.66-5.74)	0.224	1.11 (0.77-1.61)	0.576	0.92 (0.67-1.25)	0.580
Model 2	1.15 (0.85-1.56)	0.353	1.70 (0.56-5.14)	0.348	1.09 (0.75-1.59)	0.654	0.92 (0.66-1.26)	0.593
	**TSH (per μIU/mL)**		**FT4 (per ng/dL)**		**FT3 (per pg/mL)**		**FT3/FT4 ratio (per unit)**	
	**β (95% CI)**	**P Value**	**β (95% CI)**	**P Value**	**β (95% CI)**	**P Value**	**β (95% CI)**	P Value
**Outcomes**								
Total cholesterol, mg/dL								
Unadjusted model	2.17 (-4.08 to 8.41)	0.496	**33.18 (10.26 to 56.10)**	**0.005**	-3.76 (-11.57 to 4.06)	0.346	**-8.07 (-14.51 to -1.63)**	**0.014**
Model 1	0.67 (-5.64 to 6.99)	0.835	**31.96 (9.01 to 54.90)**	**0.006**	-2.83 (-10.72 to 5.07)	0.482	**-7.54 (-14.06 to -1.01)**	**0.024**
Model 2	0.61 (-5.69 to 6.91)	0.850	**32.70 (9.59 to 55.81)**	**0.006**	-3.43 (-11.34 to 4.48)	0.395	**-8.03 (-14.59 to -1.46)**	**0.017**
LDL cholesterol, mg/dL								
Unadjusted model	0.28 (-5.19 to 5.75)	0.920	**23.51 (3.42 to 43.61)**	**0.022**	-5.55 (-12.39 to 1.29)	0.112	**-7.65 (-13.28 to -2.02)**	**0.008**
Model 1	-0.49 (-6.05 to 5.06)	0.862	**22.27 (2.07 to 42.47)**	**0.031**	-4.81 (-11.75 to 2.13)	0.174	**-7.16 (-12.89 to -1.42)**	**0.015**
Model 2	-0.42 (-5.95 to 5.12)	0.882	**24.82 (4.52 to 45.13)**	**0.017**	-5.76 (-12.70 to 1.18)	0.103	**-8.15 (-13.90 to -2.39)**	**0.006**
HDL cholesterol, mg/dL								
Unadjusted model	1.93 (-0.21 to 4.07)	0.077	-3.14 (-11.08 to 4.79)	0.437	-2.17 (-4.86 to 0.51)	0.113	-0.75 (-2.98 to 1.47)	0.505
Model 1	0.43 (-1.61 to 2.48)	0.677	-3.22 (-10.73 to 4.30)	0.401	-2.01 (-4.58 to 0.56)	0.125	-0.73 (-2.86 to 1.41)	0.503
Model 2	0.40 (-1.59 to 2.40)	0.691	-1.61 (-9.01 to 5.78)	0.669	-1.77 (-4.29 to 0.74)	0.166	-0.87 (-2.97 to 1.23)	0.414
Triglycerides, mg/dL								
Unadjusted model	-0.01 (-0.09 to 0.06)	0.707	**0.50 (0.22 to 0.77)**	**<0.001**	0.06 (-0.04 to 0.15)	0.233	-0.05 (-0.13 to 0.03)	0.190
Model 1	0.00 (-0.07 to 0.08)	0.968	**0.49 (0.21 to 0.76)**	**0.001**	0.06 (-0.03 to 0.16)	0.184	-0.05 (-0.13 to 0.03)	0.242
Model 2	0.00 (-0.07 to 0.07)	0.980	**0.40 (0.13 to 0.66)**	**0.004**	0.06 (-0.03 to 0.15)	0.176	-0.03 (-0.11 to 0.04)	0.405

Model 1: age, sex. Model 2: age, sex, BMI, diabetes, smoking. Analysis of lipid profile restricted to participants not treated with lipid-lowering therapy drugs. Linear regression models were used to evaluate the associations of TSH, FT3, FT4 and FT3/FT4 ratio (independent variables) and lipid profile (dependent variables). Logistic regression models were used to evaluate the associations of TSH, FT3, FT4 and FT3/FT4 ratio (independent variables) and dyslipidemia (dependent variables). TSH and triglycerides were log-transformed. LDL, low-density lipoproteins; HDL, high-density lipoproteins; TSH, Thyroid-stimulating hormone; FT4, free thyroxine; FT3, free triiodothyronine. Significant associations are highlighted in bold.

Higher levels of FT4 and lower FT3/FT4 ratio were associated with a higher prevalence of hypertension and chronic kidney disease in unadjusted models, but not in the adjusted models (**Table 5**). Both systolic and diastolic blood pressure were not significantly associated with thyroid hormone levels. On the other hand, a lower TSH, a higher FT3 and a higher FT3/FT4 ratio were associated with higher eGFR in unadjusted and adjusted analysis (**Table 5**).

**Table 5:** Association of thyroid function with hypertension, systolic and diastolic blood pressure, kidney function, and chronic kidney disease.

	TSH (per μIU/mL)		FT4 (per ng/dL)		FT3 (per pg/mL)		FT3/FT4 ratio (per unit)	
	Odds ratio(95% CI)	P Value	Odds ratio(95% CI)	P Value	Odds ratio(95% CI)	P Value	Odds ratio(95% CI)	P Value
**Outcomes**								
Hypertension								
Unadjusted	1.12 (0.84-1.49)	0.453	**4.33 (1.48-12.68)**	**0.008**	0.84 (0.59-1.20)	0.339	**0.68 (0.51-0.93)**	**0.014**
Model 1	1.03 (0.76-1.40)	0.732	2.94 (0.94-9.17)	0.064	1.13 (0.77-1.67)	0.532	0.88 (0.64-1.22)	0.450
Model 2	1.04 (0.75-1.43)	0.828	2.93 (0.89-9.62)	0.077	1.06 (0.70-1.58)	0.794	0.83 (0.59-1.17)	0.299
CKD								
Unadjusted	1.36 (0.86-2.16)	0.191	**9.73 (1.79-52.95)**	**0.009**	**0.49 (0.27-0.90)**	**0.021**	**0.39 (0.23-0.68)**	**<0.001**
Model 1	1.47 (0.88-2.46)	0.145	4.14 (0.66-25.88)	0.128	0.75 (0.39-1.44)	0.386	0.62 (0.35-1.10)	0.100
Model 2	1.52 (0.90-2.56)	0.115	4.07 (0.64-25.92)	0.137	0.73 (0.37-1.41)	0.342	0.61 (0.34-1.09)	0.093
	**TSH (per μIU/mL)**		**FT4 (per ng/dL)**		**FT3 (per pg/mL)**		**FT3/FT4 ratio (per unit)**	
	**β (95% CI)**	**P Value**	**β (95% CI)**	**P Value**	**β (95% CI)**	**P Value**	**β (95% CI)**	**P Value**
**Outcomes**								
Systolic pressure, mmHg								
Unadjusted model	0.74 (-2.07 to 3.56)	0.604	9.08 (-1.28 to 19.44)	0.086	-0.29 (-3.80 to 3.22)	0.871	-1.73 (-4.68 to 1.22)	0.251
Model 1	0.27 (-2.40 to 2.93)	0.844	3.37 (-6.40 to 13.14)	0.499	2.98 (-0.36 to 6.31)	0.080	1.42 (-1.42 to 4.25)	0.327
Model 2	0.26 (-2.33 to 2.86)	0.843	3.06 (-6.50 to 12.62)	0.530	2.31 (-0.95 to 5.57)	0.165	0.96 (-1.81 to 3.73)	0.497
Diastolic pressure, mmHg								
Unadjusted model	1.57 (-0.04 to 3.18)	0.056	0.97 (-4.98 to 6.92)	0.750	1.07 (-0.94 to 3.09)	0.294	0.62 (-1.08 to 2.31)	0.475
Model 1	1.45 (-0.17 to 3.07)	0.079	2.00 (-3.94 to 7.94)	0.510	0.60 (-1.43 to 2.63)	0.560	0.10 (-1.62 to 1.83)	0.906
Model 2	1.46 (-0.09 to 3.01)	0.066	2.31 (-3.42 to 8.05)	0.429	-0.05 (-2.00 to 1.91)	0.964	-0.42 (-2.08 to 1.24)	0.620
eGFR, mL/min/1.73 m^2^								
Unadjusted model	**-3.28 (-5.35 to -1.22)**	**0.002**	**-9.21 (-16.88 to -1.54)**	**0.019**	**6.63 (4.07 to 9.19)**	**<0.001**	**6.05 (3.90 to 8.20)**	**<0.001**
Model 1	**-2.70 (-4.43 to -0.97)**	**0.002**	-2.12 (-8.52 to 4.28)	0.516	**2.87 (0.69 to 5.05)**	**0.010**	**2.41 (0.56 to 4.25)**	**0.011**
Model 2	**-2.72 (-4.45 to -0.99)**	**0.002**	-1.85 (-8.28 to 4.57)	0.571	**2.76 (0.57 to 4.94)**	**0.013**	**2.30 (0.44 to 4.15)**	**0.015**

Model 1: age, sex. Model 2: age, sex, BMI, diabetes, smoking (diabetes not included in model for diabetes). Analysis of fasting plasma glucose and HOMA-IR restricted to participants not treated with antidiabetic drugs. Linear regression models were used to evaluate the associations of TSH, FT3, FT4 and FT3/FT4 ratio (independent variables) and blood pressure, kidney function (dependent variables). Logistic regression models were used to evaluate the associations of TSH, FT3, FT4 and FT3/FT4 ratio (independent variables) and hypertension and CKD (dependent variables). TSH was log-transformed. CKD, chronic kidney disease; eGFR, estimated glomerular filtration rate; TSH, Thyroid-stimulating hormone; FT4, free thyroxine; FT3, free triiodothyronine. Significant associations are highlighted in bold.

Lower FT3, lower FT3/FT4 ratio and higher FT4 were associated with a higher estimated 10-year risk of cardiovascular risk events (**Supplementary Table 1**) and a higher cardiovascular risk category (**Table 6**). The proportion of participants in the very high risk category was higher in the lower tercile of FT3 (32.6% vs 21.7% in the higher tercile), in the lower tercile of FT3/FT4 ratio (30.2% vs 17.6% in the higher tercile) and in the upper tercile of FT4 (29.8% vs 21.4% in the lower tercile). An inverse distribution was observed in the low-moderate risk category (**Table 6**).

**Table 6:** Association of thyroid function with cardiovascular risk categories.

TSH				FT4			
Cardiovascular risk category	Lower tercile(0.36-1.02 μIU/mL)	Middle tercile(1.03-1.52 μIU/mL)	Higher tercile(1.53-4.86 μIU/mL)	Cardiovascular risk category	Lower tercile(0.74-1.01 ng/dL)	Middle tercile(1.02-1.12 ng/dL)	Higher tercile(1.13-1.46 ng/dL)
Low-moderate risk	40.5%	40.8%	44.4%	Low-moderate risk	47.7%	42.0%	35.1%
High risk	31.9%	33.2%	32.1%	High risk	30.9%	31.5%	35.1%
Very high risk	27.6%	26.0%	23.5%	Very high risk	21.4%	26.5%	29.8%
FT3				FT3/FT4 ratio			
**Cardiovascular risk category**	**Lower tercile (1.71-2.33 pg/mL)**	**Middle tercile (2.34-2.70 pg/mL)**	**Higher tercile (2.71-3.69 pg/mL)**	**Cardiovascular risk category**	**Lower tercile (1.35-2.15)**	**Middle tercile (2.16-2.55)**	**Higher tercile (2.56-4.18)**
Low-moderate risk	38.1%	41.0%	46.9%	Low-moderate risk	37.3%	37.4%	50.8%
High risk	29.3%	36.2%	31.4%	High risk	32.5%	33.1%	31.6%
Very high risk	32.6%	22.8%	21.7%	Very high risk	30.2%	29.5%	17.6%

Cardiovascular disease risk categories were based on SCORE2 and SCORE2-OP according to the European Society of Cardiology classification: low-moderate risk (<50 years: risk <2.5%; 50–69 years: <5%; ≥70 years: <7.5%), high risk (<50 years: 2.5-7.5%; 50–69 years: 5-10%; ≥70 years: 7.5-15%) or very high risk (<50 years: ≥7.5%; 50–69 years: ≥10%; ≥70 years: ≥15%). P for trend across terciles - TSH: p=0.278; FT4: p=0.004; FT3: p=0.009; FT4/FT3 ratio: p<0.001. TSH, Thyroid-stimulating hormone; FT4, free thyroxine; FT3, free triiodothyronine. Significant associations are highlighted in bold.

## Discussion

In a population-based study, we observed a significant association of thyroid function within the euthyroid range with cardiovascular risk factors. On one hand, participants with higher levels of FT3 and/or FT3/FT4 ratio had higher BMI, fat mass and waist circumference, and higher levels of hs-CRP. On the other hand, participants with lower FT3/FT4 ratio and/or higher FT4 levels had higher levels of LDL cholesterol and triglycerides, and higher prevalence of diabetes. Lower FT3 levels and FT3/FT4 ratio, and higher TSH levels were associated with decreased kidney function. Furthermore, lower FT3, lower FT3/FT4 ratio and higher FT4 were associated with an increased 10-year estimated risk of cardiovascular events.

Thyroid hormones play an important role in regulating metabolic rate and body composition (2). Hyperthyroidism promotes increased metabolic rate and weight loss, whereas hypothyroidism promotes weight gain (4, 5). Less well-defined is the association between thyroid function and body composition in euthyroid individuals. We observed that participants with higher BMI, fat mass and waist circumference had higher levels of FT3 and FT3/FT4 ratio, which is in agreement with previous studies (36–38). The fat mass directly increases type 1 and 2 deiodinase activity increasing the peripheral conversion of T4 in T3 (39). Higher levels of FT3 can be interpreted as an adaptive process to ameliorate obesity-related morbidities (40, 41), as thyroid hormone effects may limit nutrient overload by inducing tissue thermogenesis and by stimulating metabolic activity (39). The higher FT3/FT4 ratio with increasing BMI may also be a marker of obesity-induced thyroid hormone resistance (42, 43). Laclaustra et al. showed that thyroid hormone resistance is associated with worse cardiometabolic parameters (44). We also found a positive association between TSH and leptin, even after adjustment for confounders including BMI. The relationship between thyroid axis and leptin is complex (43, 45, 46). On one hand, leptin actives the TRH-TSH-thyroid gland axis at a central level; on the other hand, TSH may stimulate leptin secretion by a direct effect on adipocytes (47, 48). Whether this relationship is adaptative or maladaptive in obesity is unsettled (2, 43).

In our study, higher hs-CRP levels were associated with higher levels of FT3 and FT3/FT4 ratio. Jublanc et al. observed a negative correlation of hs-CRP with FT4 (49), and Roef et al. described higher hs-CRP and IL-6 levels to be associated with higher FT3, lower FT4, and a higher FT3/FT4 ratio (50). Most studies in overt or subclinical hyperthyroidism have not found an association between thyroid function and systemic inflammation (51, 52). Thus, we hypothesize that higher hs-CRP levels in euthyroid individuals with higher levels of FT3 and FT3/FT4 ratio are a marker of thyroid hormone resistance and not a consequence of increased thyroid hormone effects.

Concerning the glycemic and lipid profiles, the association of higher levels of FT4 with diabetes and worse lipid profile in the absence of associations with FT3 and TSH suggests that the increase in FT4 represents a decrease in the conversion to the active form T3, making it unlikely to represent an increased thyroid function. The fact that a lower FT3/FT4 ratio was associated with diabetes and worse lipid profile further reinforces that a decrease of the peripheral conversion of T4 to T3 may contribute to metabolic dysfunction. Despite the known role of overt hypothyroidism in dyslipidemia and diabetes, the effect of subclinical hypothyroidism and thyroid hormones within the euthyroid range remains uncertain (53–55). In a population at high cardiovascular risk, de Vries et al. found no association between TSH levels in the euthyroid range and incident diabetes (55). On the other hand, in a population-based prospective cohort, Chaker et al. showed that low and low-normal thyroid function are risk factors for incident diabetes (16). Garduño-García et al. also showed that low thyroid function, even in the euthyroid range, predisposes to higher cholesterol, glucose and HOMA-IR levels (56). Our results further highlight a potential role of low-normal levels of thyroid hormones in increasing the risk of diabetes and dyslipidemia.

Regarding kidney function, thyroid hormones directly affect renal hemodynamics, and sodium and water homeostasis (57). More specifically, thyroid hormones increase kidney blood flow and activate renin-angiotensin-aldosterone system, therefore, increasing glomerular filtration rate (57). In this study, we found an association between lower thyroid function (higher TSH and lower FT3) and decreased glomerular filtration rate. In agreement with our findings, an observational study of 309 patients with stage 2-4 chronic kidney disease and subclinical hypothyroidism observed a preservation of renal function and decreased risk of adverse renal outcomes in those treated with levothyroxine (58). These results are important because decreased glomerular filtration rate has been recognized as a major risk factor for cardiovascular disease (59) and thyroid hormones may directly modulate it.

Regarding cardiovascular risk, lower FT3, lower FT3/FT4 ratio and higher FT4 levels were associated with an increased 10-year estimated risk of cardiovascular events. This is in agreement with previous studies that showed that individuals with lower FT3 levels within the euthyroid range had increased cardiovascular mortality (17) and all-cause mortality (60, 61). A higher risk of mortality has also been described for individuals with higher FT4 (62) and lower FT3/FT4 ratio (63), supporting our observation of an increased risk of cardiovascular events in these groups. The more adverse lipid profile with lower FT3 and higher FT4 probably contributed to the increased risk calculated by the SCORE2 and SCORE2-OP. These results remained significant even after adjustment for BMI which is not included in the risk score. Furthermore, lower FT3, lower FT3/FT4 ratio and higher FT4 are also associated with a higher prevalence of diabetes and lower eGFR which suggests that the risk of cardiovascular events in these groups may be even higher than that calculated with SCORE2/SCORE2-OP. Whether the risks associated with a lower FT3/FT4 ratio represent a causal effect of decreased deiodinase activity or simply the combination of the risk associated with a low FT3 (marker of frailty and/or pro-inflammatory state) and a higher FT4 (which may have some direct effects and/or represent a better marker of thyroid effects on some tissues) is uncertain; as an example, previous studies have suggested that T4 may be a better marker of thyroid hormone action on the risk of development of atrial fibrillation (64, 65). Our results suggest that reference values for thyroid hormone levels may have to account for cardiovascular risk. Furthermore, future studies should evaluate if thyroid hormone levels can be used to improve cardiovascular risk prediction.

In our study, most associations of thyroid function with cardiovascular risk factors were observed only for FT4, FT3 or FT3/FT4 ratio, and not for TSH. A recent meta-analysis of 58 studies also described that thyroid hormone levels, particularly FT4 levels, were more strongly associated with clinical parameters than TSH (66). Our results also support that, within the euthyroid range, thyroid hormone levels are more correlated with cardiovascular risk factors than TSH levels.

Our results are clinically relevant as they suggest that mild variations of thyroid hormones within the euthyroid range may modulate cardiovascular risk in the general population. Our study comprised a large sample of individuals aged 45 years or older allowing us to represent the segment of the population usually targeted for cardiovascular preventive interventions. The use of a comprehensive panel of thyroid tests is also a strength, as previous studies evaluating the association of thyroid function with cardiovascular risk factors focused mainly on the impact of TSH and FT4. Also, we included an elaborated panel of cardiometabolic parameters, which allowed us to have an in-depth insight into the association between endocrine and cardiovascular systems. We evaluated not only individual risk factors, but also the global cardiovascular risk which is more relevant from a clinical perspective. Furthermore, this is one of the first studies to use the recently published SCORE2/SCORE2-OP to evaluate the global cardiovascular risk, which is the most robust cardiovascular risk score currently available for European populations.

There are limitations that should also be acknowledged. First, the cross-sectional nature of the study limits the ability to draw conclusions on causation. Second, thyroid function has an important inter-individual variation that may have underestimated the associations we found (67); in addition, thyroid function was assessed at a single time point for each participant, and acute illness or other interfering factors may transiently affect thyroid hormone levels. Third, despite the differences in TSH distribution according to age, we used a single TSH reference range since these alterations are highly variable among individuals (68). We only evaluated the free thyroid hormones concentration, which are considered to represent the biologically active fraction of thyroid hormones (69). Different associations could have been found if we had evaluated total thyroid hormones concentration. Furthermore, we did not evaluate the association of surrogate markers of low thyroid hormone tissue levels (22, 70) with cardiovascular risk factors. Also, since it was not possible to measure anti-thyroid antibodies in frozen serum samples, we cannot evaluate the influence of thyroid autoimmunity on cardiovascular risk factors. We evaluated the association of thyroid function with multiple outcomes which increases the probability of obtaining significant results due to chance; given the exploratory nature of our study, no adjustment for multiple comparison was performed. Our results on the association of thyroid function with cardiovascular risk were based on a prediction model and not on cardiovascular events. Finally, despite the adjustment for main known confounders, we cannot exclude the presence of residual confounding (e.g., related to diet or exercise).

In conclusion, in a population-based study of individuals aged 45 years or older, variations of thyroid function within the euthyroid range were associated with cardiovascular risk factors. On one hand, individuals with higher BMI, larger waist circumference and higher hs-CRP had higher levels of FT3 and FT3/FT4 ratio. On the other hand, a decreased conversion of T4 to T3 (lower FT3, lower FT3/FT4 ratio and/or higher FT4) was associated with a higher prevalence of diabetes, a more adverse lipid profile, a lower eGFR and an increased 10-year estimated risk of cardiovascular events. Further studies, ideally randomized clinical trials, are needed to evaluate if the modulation of thyroid hormones axis may be a therapeutic target to decrease cardiovascular risk.

## Data availability statement

The datasets presented in this article are not readily available because the dataset used in this analysis is managed by Instituto de Saúde Pública da Universidade do Porto (ISPUP). Requests to access the datasets should be directed to https://ispup.up.pt/en/cohorts.

## Ethics statement

The studies involving human participants were reviewed and approved by Ethics committee of Centro Hospitalar Universitário São João/Faculdade de Medicina da Universidade do Porto. The patients/participants provided their written informed consent to participate in this study.

## Author contributions

JN, MB-C, AL, MH and CV contributed to the conception and design of this work, the statistical analysis, and interpretation of data. RF-C, SM, JG, DC, AA and AL-M contributed to interpretation of data. JN, MB-C, MH, CV, and AL prepared the first draft of this paper. All authors contributed to the article and approved the submitted version.

## Funding

This work was supported by the DOCnet project (NORTE-01-0145-FEDER-000003), supported by Norte Portugal Regional Operational Program (NORTE 2020), under the PORTUGAL2020 Partnership Agreement, through the European Regional Development Fund (ERDF), and the NETDIAMOND project (POCI-01-0145-FEDER-016385), supported by European Structural and Investment Funds, Lisbon’s Regional Operational Program 2020, and national funds from the Portuguese Foundation for Science and Technology – both projects through the Cardiovascular Research Center (UnIC, FCT 51/94) – and by the Portuguese Foundation for Science and Technology (grant POCI/SAU-ESP/61492/2004) and the Unidade de Investigação em Epidemiologia – Instituto de Saúde Pública da Universidade do Porto (EPIUnit) (POCI-01-0145-FEDER-006862, ref. UID/DTP/04750/2013). This work is financed by national funds through the FCT - Foundation for Science and Technology, I.P., within the scope of projects UIDB/04750/2020 and LA/P/0064/2020.

## Conflict of interest

The authors declare that the research was conducted in the absence of any commercial or financial relationships that could be construed as a potential conflict of interest.

## Publisher’s note

All claims expressed in this article are solely those of the authors and do not necessarily represent those of their affiliated organizations, or those of the publisher, the editors and the reviewers. Any product that may be evaluated in this article, or claim that may be made by its manufacturer, is not guaranteed or endorsed by the publisher.
