# Supplementary material for: Association of thyroid function, within the euthyroid range, with cardiovascular risk: The EPIPorto study
Source: Front Endocrinol (Lausanne). 2022 Nov 28;13:1067801. doi: 10.3389/fendo.2022.1067801 (PMC9742360; doi:10.3389/fendo.2022.1067801)
Supplement: Supplementary file 1 [file Table_1.docx]

Supplementary Material

| **Supplementary Table 1. Association of thyroid function with 10-year risk of cardiovascular disease (SCORE2 and SCORE2-OP)** | | | | | | | | |
| --- | --- | --- | --- | --- | --- | --- | --- | --- |
|  | TSH (per μIU/mL) | | FT4 (per ng/dL) | | FT3 (per pg/mL) | | FT3/FT4 ratio (per unit) | |
|  | β (95% CI) | P Value | β (95% CI) | P Value | β (95% CI) | P Value | β (95% CI) | P Value |
| **Outcomes** |  |  |  |  |  |  |  |  |
| 10-year risk of cardiovascular disease | | |  |  |  |  |  |  |
| Unadjusted | -0.07 (-0.20 to 0.06) | 0.277 | **0.78 (0.30 to 1.26)** | **0.002** | **-0.28 (-0.44 to -0.12)** | **0.001** | **-0.33 (-0.46 to -0.19)** | **<0.001** |
| Adjusted | -0.07 (-0.20 to 0.06) | 0.287 | **0.77 (0.29 to 1.25)** | **0.002** | **-0.29 (-0.45 to -0.14)** | **<0.001** | **-0.34 (-0.47 to -0.20)** | **<0.001** |
| Adjusted model: BMI. Linear regression models were used to evaluate the associations of TSH, FT3, FT4 and FT3/FT4 ratio (independent variables) with 10-year risk of cardiovascular disease (dependent variables). Patients with diabetes were excluded from these analyses. 10-year risk of fatal and non-fatal cardiovascular events was calculated using SCORE2 and SCORE2-OP. TSH and the 10 year risk of cardiovascular events were log-transformed. TSH: Thyroid-stimulating hormone; FT4: free thyroxine; FT3: free triiodothyronine. SCORE2: Systematic Coronary Risk Estimation 2; SCORE2-OP: Systematic Coronary Risk Estimation 2-Older Persons. | | | | | | | | |
